# Polymorphisms of the *pfmdr1 *but not the *pfnhe-1 *gene is associated with *in vitro *quinine sensitivity in Thai isolates of *Plasmodium falciparum*

**DOI:** 10.1186/1475-2875-11-7

**Published:** 2012-01-05

**Authors:** Teera Poyomtip, Nantana Suwandittakul, Narumon Sitthichot, Rommanee Khositnithikul, Peerapan Tan-ariya, Mathirut Mungthin

**Affiliations:** 1 Department of Biology, Faculty of Science, Mahidol University, Bangkok 10400, Thailand; 2 Department of Parasitology, Phramongkutklao College of Medicine, 315 Ratchawithi Rd., Ratchathewi, Bangkok 10400, Thailand; 3 Department of Microbiology, Faculty of Science, Mahidol University, Bangkok 10400, Thailand

## Abstract

**Background:**

The emergence of *Plasmodium falciparum *resistance to most currently used anti-malarial drugs is a major problem in malaria control along the Thai-Myanmar and Thai-Cambodia borders. Quinine (QN) with tetracycline/doxycycline has been used as the second-line treatment for uncomplicated falciparum malaria. In addition, QN monotherapy has been the first-line treatment for falciparum malaria in pregnant women. However, reduced in vitro and in vivo responses to QN have been reported. To date, a few genetic markers for QN resistance have been proposed including *Plasmodium falciparum *chloroquine resistance transporter (*pfcrt*), *P. falciparum *multidrug resistance 1 (*pfmdr1*), and *P. falciparum Na^+^/H^+ ^exchanger *(*pfnhe-1*). This study was to investigate the role of the *pfmdr1 *and *pfnhe-1 *gene on *in vitro *QN sensitivity in Thai isolates of *P. falciparum*.

**Methods:**

Eighty-five Thai isolates of *P. falciparum *from the Thai-Myanmar and Thai-Cambodia borders from 2003-2008 were determined for *in vitro *QN sensitivity using radioisotopic assay. Polymorphisms of the *pfmdr1 *and *pfnhe-1 *gene were determined by PCR-RFLP and sequence analysis. Associations between the *in vitro *QN sensitivity and the polymorphisms of the *pfmdr1 *and *pfnhe-1 *gene were evaluated.

**Results:**

The mean QN IC_50 _was 202.8 nM (range 25.7-654.4 nM). Only four isolates were QN resistant when the IC_50 _of >500 nM was used as the cut-off point. Significant associations were found between the *pfmdr1 *mutations at codons N86Y and N1042D and *in vitro *QN sensitivity. However, no associations with the number of DNNND, DDNNNDNHNDD, and NHNDNHNNDDD repeats in the microsatellite ms4760 of the *pfnhe-1 *gene were identified.

**Conclusion:**

Data from the present study put doubt regarding the *pfnhe-1 *gene as to whether it could be used as the suitable marker for QN resistance in Thailand. In contrast, it confirms the influence of the *pfmdr1 *gene on *in vitro *QN sensitivity.

## Background

The emergence of anti-malarial resistance in *Plasmodium falciparum *is a major public health threat worldwide, especially in tropical developing countries. The situation of multidrug-resistant falciparum malaria is most serious along the Thai-Myanmar and Thai-Cambodia borders [1]. To handle this situation, WHO recommends artemisinin derivative-based combination treatment (ACT) for the treatment of uncomplicated falciparum malaria [2]. Artesunate-mefloquine combination has been used as the first-line treatment in Thailand for more than 15 years [3]. Quinine (QN)-tetracycline/doxycycline has been used as the second-line treatment for uncomplicated falciparum malaria in Thailand. In addition, QN monotherapy is the first-line treatment for pregnancy [3,4]. Unfortunately reduced *in vitro *and *in vivo *response to QN has been reported in Southeast Asia [5,6]. Investigations have been carried out to identify the mechanisms of QN resistance. At least three candidate genes including *Plasmodium falciparum *chloroquine resistance transporter (*pfcrt*), *Plasmodium falciparum *multidrug resistance 1 (*pfmdr1*), and *Plasmodium falciparum Na^+^/H^+ ^exchanger *(*pfnhe-1*) have been linked to reduced QN sensitivity [7-12]. Cooper *et al*. (2002) showed the association between the *pfcrt *mutation at codon 76 and QN sensitivity [7]. In addition, recent studies have shown that parasites containing a novel mutation in the *pfcrt *gene, Q352K/R, C350R, altered QN sensitivity [8,9]. Concerning the *pfmdr1 *gene, it has been shown that both mutations and copy number influenced *in vitro *QN sensitivity [10-12]. Using quantitative trait loci on the genetic cross of HB3 and Dd2 strains, an additional candidate gene, *pfnhe-1*, for QN resistance was identified [13]. This gene encodes a 226 kDa parasite plasma membrane protein containing 12 transmembrane domains, and 3 microsatellite regions, msR1, ms3580 and ms4760. Variations in QN susceptibilities between different parasite strains have also been linked to repeat polymorphisms in the microsatellite locus ms4760 of *pfnhe-1 *[13]. A few studies showed that the number of DNNND, DDNNNDNHNDD, and NHNDNHNNDDD repeats in the microsatellite ms4760 influenced *in vitro *QN sensitivity [14-19]. However, there is a lack of consensus regarding the specific nature of these associations [20-25]. For instance, while some studies report an association between reduced susceptibility to QN and an increase in the number of 'DNNND' repeats in ms4760 [15,18,19], others could not verify this association [22-24], or found that two DNNND repeats was the optimal number for conferring a reduction in QN sensitivity [17]. Moreover, amplification of a second ms4760 repeat 'NHNDNHNNDDD' has been linked to increases in the parasite's susceptibility to QN [15,18,19]. However, the reverse association has also been reported [22], and several studies have failed to confirm either of these findings [23,24]. Considering the situation of multidrug-resistant *P. falciparum *in Thailand, determination of the molecular basis of QN resistance is crucial to be able to monitor parasite resistance. This study was to investigate the influence of the *pfmdr1 *and *pfnhe-1 *genes on *in vitro *QN sensitivity of Thai isolates of *P. falciparum *from both the Thai-Myanmar and Thai-Cambodia borders.

## Methods

### *Plasmodium falciparum *strains and cultivation

The 85 isolates of *P. falciparum *used in this study were collected from patients with uncomplicated falciparum malaria, who attended malaria clinics and hospitals in malaria endemic areas along the Thai-Myanmar (Kanchanaburi and Ranong) and Thai-Cambodia (Chantaburi and Srisaket) borders from 2003 to 2008. The research protocol was reviewed and approved by the Ethics Committee of the Royal Thai Army Medical Department. Parasites were maintained in continuous cultures using a modification of the method of Trager and Jensen [26].

### *In vitro *sensitivity assays

QN sensitivity of *P. falciparum *isolates was determined by measurement of [3H] hypoxanthine incorporation into parasite nucleic acids as previously described [27]. Drug IC_50 _(i.e. concentration of a drug which inhibits parasite growth by 50%) was determined from the log dose/response relationship as fitted by GRAFIT (Erithacus Software, Kent, England).

### Genotypic characterization for *pfmdr1 *and *pfnhe-1 *genes

Parasite DNA was extracted using the Chelex-resin method [28]. Five microliters of DNA preparation was used for a 25 μl PCR reaction. Mutations in the *pfmdr1 *gene were determined by the nested PCR and restriction endonuclease digestion method developed by Duraisingh *et al*. for detection of the mutations at codons 86, 184, 1034, 1042 and 1246. K1 and 7G8 strain were used as positive controls [29]. The *pfmdr1 *gene copy number was determined by TaqMan real-time PCR (ABI sequence detector 7000; Applied Biosystems) as developed by Price *et al*. [30]. The K1 and Dd2 clone containing 1 and 4 *pfmdr1 *copies, respectively was used as the reference DNA sample. The *pfmdr1 *and β-tubulin amplification reactions were run in duplicate. Relative *pfmdr1 *copy number was assessed as previously described. PCR amplification for the *pfnhe-1 *ms4760 microsatellite was performed as previously described [14]. DNA purification and DNA sequencing were conducted by Bioservice Unit, Bangkok, Thailand. Sequences were analyzed for the number of DNNND, DDNNNDNHNDD, and NHNDNHNNDDD repeats in the *pfnhe-1 *ms4760 microsatellite by BioEdit sequence alignment editor (version 7.0.9.0).

### Statistical analysis

Data were analysed by SPSS for Windows version 18 (SPSS Inc., Chicago, IL). The SPSS license number is ID5071846. The QN IC_50 _of each isolate was the mean IC_50 _of three independent experiments. Each experiment was carried out in triplicate. Normally distributed IC_50 _data were assessed by the Kolmogorov Smirnov test. Differences among parasites with different genotypes was analysed by Chi square and Fisher's exact test. Correlations between QN IC_50 _and the *pfmdr1 *copy number and the number of DNNND, DDNNNDNHNDD, and NHNDNHNNDDD repeats in the *pfnhe-1 *ms4760 microsatellite were assessed by Pearson's correlation. Differences of the mean QN IC_50 _among parasites from different groups were analyzed by Independent *t *test or One-way ANOVA. Post Hoc test (Scheffe) for multiple comparisons was used to test for differences among groups. The level of significance was set at a *p *value of <0.05.

## Results

### *In vitro *QN sensitivity

Characteristics of parasite isolates are presented in Table 1. The mean IC_50 _(± SD) for QN was 202.8 ± 123.3 nM (range 25.7-654.4 nM). QN IC_50_s in this population of isolates were normally distributed. No significant differences were found between QN IC_50 _of parasites isolated from the Thai-Cambodia and Thai-Myanmar borders (*p *= 0.641, Independent *t *test). Of 85 isolates, only 4 (4.7%) isolates exhibited QN IC_50 _of >500 nM. Characterization of the four QN-resistant *P*. *falciparum *isolates is shown in Table 2.

**Table 1:** *In vitro *sensitivity to QN and distribution of *pfmdr1 *polymorphisms of the 85 adapted parasites from Thai-Myanmar and Thai-Cambodia areas

Area	**No**.	Mean QN	Mean	*pfmdr*1 mutations				
		**IC_50 _(nM)**	***pfmdr*1 copy number**	**86Y**	**184F**	**1034C**	**1042D**	**1246Y**

Thai- Myanmar	37	209.9 ± 117.1	2.9 ± 1.4*	6 (16.2%)	15 (40.5%)	4 (10.8%)	4 (10.8%)	-

Thai- Cambodia	48	197.2 ± 128.9	1.2 ± 0.7	9 (18.8%)	38 (79.2%)**	10 (20.8%)	13 (24.1%)	-

Total	85	202.8 ± 123.3	2.0 ± 1.3	15 (17.6%)	53 (61.4%)	14 (16.5%)	17 (20.0%)	-

* Significant difference between two areas determined by Independent *t *test (*p *< 0.001)

** Significant difference between two areas determined by Chi square test (*p *< 0.001)

**Table 2:** Characterization of the four QN-resistant isolates of *Plasmodium falciparum*

	SK20	SK22	MR2	KB12
**Origin**	**Srisaket**	**Srisaket**	**Chantaburi**	**Kanchanaburi**

QN IC_50 _(nM)	521.8	503.0	530.6	654.4

*Pfmdr1 *mutations				

N86Y	N	N	N	N

Y184F	F	F	F	F

S1034C	S	S	S	C

N1042D	N	N	N	D

D1246Y	D	D	D	D

*Pfmdr1 *copy number	0.8	0.9	1.2	2.0

*Pfnhe-1*				

DNNND repeats	3	4	1	3

DDNNNDNHNDD repeats	1	2	1	1

NHNDNHNNDDD repeats	1	1	0	1

### Characterization of the *pfmdr1 *and *pfnhe-1 *genes

Characterization of the *pfmdr1 *gene of 85 isolates is shown in Table 1. Approximately 60% of the parasite isolates contained the *pfmdr1 *184F allele. Determination of the *pfmdr1 *gene copy number showed that these isolates contained *pfmdr1 *copy numbers with a mean of 2.0 (range 0.7-5.6). The *pfmdr1 *184F allele was more common in the parasites isolated from the Thai-Cambodia border compared with those from the Thai-Myanmar border. In contrast, the parasites isolated from the Thai-Myanmar border had significantly higher copy numbers. A total of 81 isolates were characterized for polymorphisms in the number of DNNND, DDNNNDNHNDD, and NHNDNHNNDDD repeats in the *pfnhe-1 *ms4760 microsatellite (Table 3). The most common number of DNNND, DDNNNDNHNDD, and NHNDNHNNDDD repeats was 4 (48.2%), 2 (79%) and 1 (86.4%). Genotypic characterization of the four QN-resistant isolates is shown in Table 2.

**Table 3:** Comparison of *in vitro *QN sensitivity among *Plasmodium falciparum *with different *pfmdr1*and *pfnhe-1 *genotypes

Parasite genotypes		No. (%)	Mean QN IC_50_	*p *value
			**(nM)**	

*pfmdr1*				

86	N86	70 (82.4)	216.5 ± 127.5	0.025*

	86Y	15 (17.6)	138.3 ± 76.1	

184	Y184	32 (37.7)	160.4 ± 93.6	0.013*

	184F	53 (62.3)	228.3 ± 132.6	

1034	S1034	71 (83.5)	194.1 ± 114.0	0.144

	1034C	14 (16.5)	246.9 ± 160.5	

1042	N1042	68 (80)	185.8 ± 113.8	0.010*

	1042D	17 (20)	270.5 ± 139.9	

Copy no.	<3	69 (81.2)	195.8 ± 132.2	0.284

	≥ 3	16 (18.8)	232.7 ± 68.8	

*pfnhe-1*				

DNNND repeats	0	1 (1.2)	183.6	0.958

	1	3 (3.7)	246.1 ± 147.1	

	2	5 (6.2)	163.7 ± 44.2	

	3	26 (32.1)	203.0 ± 155.3	

	4	39 (48.2)	213.0 ± 108.6	

	5	7 (8.6)	195.9 ± 92.0	

DDNNNDNHNDD repeats	0	3 (3.7)	115.7 ± 71.3	0.049**

	1	14 (17.3)	272.6 ± 193.1	

	2	64 (79.0)	195.8 ± 102.4	

NHNDNHNNDDD repeats	0	9 (11.1)	196.4 ± 137.6	0.378

	1	70 (86.4)	210.8 ± 124.2	

	2	2 (2.5)	87.0 ± 47.4	

* Significant difference determined by Independent *t *test

* Significant difference determined by One-way ANOVA

### The association between *in vitro *quinine sensitivity and the *pfmdr1 *and *pfnhe-1 *genes

No correlation was found between QN IC_50 _and the *pfmdr1 *copy number (*r *= 0.099, *p *= 0.367) and the number of DNNND (*r *= 0.017, *p *= 0.880), DDNNNDNHNDD (*r *= -0.76, *p *= 0.503), and NHNDNHNNDDD (*r *= -0.420, *p *= 0.711) repeats in the *pfnhe-1 *ms4760 microsatellite. Table 3 shows the *in vitro *QN sensitivities of these adapted Thai isolates containing different *pfmdr1 *genotypes and different number of DNNND, DDNNNDNHNDD, and NHNDNHNNDDD repeats in the *pfnhe-1 *ms4760 microsatellite. Parasite isolates with *pfmdr1 *184F and 1042D showed significantly higher QN IC_50 _than those containing *pfmdr1 *184Y and 1042N, respectively. In contrast, parasites having *pfmdr1 *86Y exhibited significantly lower QN IC_50 _than those having *pfmdr1 *86N. Parasites containing different *pfmdr1 *copy number and the number of DNNND and NHNDNHNNDDD repeats in the *pfnhe-1 *ms4760 microsatellite showed no significant difference in the mean QN IC_50_. When the parasites were categorized into subgroups according to their copy number of the *pfmdr1*gene, using 3 copies as the cut-off point gave the greatest difference of the QN IC_50 _between 2 groups compared to other figures. However no significant difference was detected. These parasites were also classified into subgroups with a different number of DNNND repeats. No significant difference were found between QN IC_50 _of parasites containing ≥2 and less repeats (204.9 ± 121.5 nM & 230.5 ± 204.1 nM, *p *= 0.692), ≥3 and less repeats (207.7 ± 124.7 nM & 207.7 ± 124.7 nM, *p *= 0.748) or ≥4 and less repeats (210.4 ± 105.5 nM & 200.5 ± 148.1 nM, *p *= 0.728). Although analysis by One-way ANOVA showed a significant difference of QN IC_50 _in the parasites with a different number of DDNNNDNHNDD repeats, multiple comparison showed no significant difference between groups.

According to their *pfmdr1 *haplotypes, parasites were classified into five groups (Table 4), i.e., the isolates containing the *pfmdr1 *86Y, 1042D, 184F with copy number <3, 184Y with copy number <3 and 184Y with copy number ≥3. Significant differences of QN IC_50 _were found among these groups (*p *= 0.019, One-way ANOVA). Multiple comparison indicated that only parasites containing *pfmdr1 *1042D were significantly less sensitive to QN than parasites containing *pfmdr1 *86Y (*p *= 0.048).

**Table 4:** Comparison of *in vitro *QN sensitivity among *Plasmodium falciparum *with different *pfmdr1 *haplotypes

Group	*Pfmdr1 *haplotypes				No. (%)	Mean QN IC_50 _(nM) ± SD	*p *value
				
	N86Y	Y184F	N1042D	**Copy No**.			
1	Y	Y/F	N	<3/≥3	15 (17.6)	138.3 ± 76.1	0.019
	
2	N	F	D	<3/≥3	17 (20.0)	270.5 ± 139.9	
	
3	N	F	N	< 3	30 (35.3)	200.4 ± 133.3	
	
4	N	Y	N	< 3	12 (14.1)	162.1 ± 113.0	
	
5	N	Y	N	≥3	11 (12.9)	236.7 ± 74.0	

Total					85 (100)	202.8 ± 123.3	

Significant difference among groups determined by One-way ANOVA

## Discussion

In the present study, using the IC_50 _of >500 nM as the cut-off point for *in vitro *QN resistance, only 4.7% (4/85) exhibited QN resistance. Although a higher cut-off point at 800 nM has been proposed, no parasite isolate was found showing QN IC_50 _of >800 nM. This result is similar to those previous reports showing that most Thai isolates of *P. falciparum *were QN sensitive [6,31,32]. However data from these studies might not be comparable since different methods including schizont maturation inhibition, isotopic and SYBR green 1 based fluorescence assays were used for the determination of QN IC_50_. In addition, since culture-adapted isolates were used in some studies including the present study, specific phenotypes might be selected during the adaptation process. The situation of QN resistance in Thailand is less serious than those found in mefloquine and chloroquine. This may be due to a lower drug pressure of QN since it has a shorter half life. However, a decline in QN sensitivity of *P. falciparum *isolated from the Thai-Myanmar border has been indicated in recent study [6].

All parasite isolates in this study contained chloroquine-resistant haplotype, CVIET of the *pfcrt*. The association between *in vitro *QN sensitivity and polymorphisms of the *pfmdr1 *gene, but not the *pfnhe-1 *gene, was identified. Although a genetic cross study indicated that QN sensitivity can be modulated by the *pfnhe-1 *gene [13], the role of the *pfnhe-1 *gene as a molecular marker for QN resistance is still controversial. Some but not all *in vitro *and *in vivo *studies identified the association between DNNND, DDNNNDNHNDD, and NHNDNHNNDDD repeats of the *pfnhe-1 *ms4760 microsatellite and QN sensitivity and treatment outcome, respectively [14-19,23-25]. Nearly half of Thai isolates in the present study contained 4 DNNND repeats while most parasites from other areas in Southeast Asia including Vietnam and the China-Myanmar border contained 3 DNNND repeats [18,19]. Similar to these 2 studies, most isolates in the present study contained 1 repeat of NHNDNHNNDDD. The studies with positive association usually showed that parasites with 2 or more than 2 DNNND repeats had a significantly reduced QN sensitivity compared with those with 1 repeat. This association has been found in the studies from Vietnam and the China-Myanmar border as well [18,19]. In contrast, no significant difference of QN IC_50 _between parasites containing ≥2 and less DNNND repeats was identified. Inconsistent findings of the association between the response to QN and *pfnhe-1 *gene might be due to its interaction with other genes such as *pfcrt *and *pfmdr1*. This postulation has been provided to explain the result of a knockdown *pfnhe-1 *expression resulting in increased QN sensitivity in 2 of 3 parasite lines [16]. In addition, a recent study in Kenya found no significant difference of QN IC_50 _among parasites with different DNNND repeats [17]. However parasites containing 2 DNNND repeats with 86Y *pfmdr1 *showed a decrease in QN sensitivity. When the QN IC_50 _of the parasites containing a similar number of DNNND repeats in the *pfnhe-1 *ms4760 microsatellite with different 86 alleles in the *pfmdr1 *gene was compared, no significant difference of QN IC_50 _among these parasites was identified (data not shown). In addition, no significant correlations between the number of DDNNNDNHNDD, and NHNDNHNNDDD repeats of the *pfnhe-1 *ms4760 microsatellite and *in vitro *QN sensitivity were found. Since there were only four isolates exhibiting reduced QN susceptibility, this may be restrictive for identification or validation of these new markers.

In contrast to the *pfnhe-1 *gene, *in vitro *QN sensitivity was significantly associated with the mutations in the *pfmdr1 *gene in these Thai isolates. The parasites containing the *pfmdr1 *184F and 1042D allele showed less sensitivity to QN while those with the *pfmdr1 *86Y exhibited increased QN sensitivity. However, when the parasites were categorized according to their haplotypes of the *pfmdr1 *gene, the *pfmdr1 *86Y and 1042D allele influenced QN sensitivity. Compelling evidence for a significant role of N1042D mutation on *in vitro *QN sensitivity has been shown in a few studies using allelic exchange strategies [10,11]. Reed *et al*. [10] showed that insertion of the *pfmdr1 *gene containing the 1034C, 1042D and 1246Y alleles made a QN - sensitive parasite become more resistant to QN [10]. Conversely, a QN -resistant line became more sensitive to QN after these alleles were removed. More recently, a study by Sidhu *et al*. (2005) identified that a single mutation, the N1042D, could modulate the parasites become less sensitive to QN [11]. Unlike the N1042D mutation, the functional role of the N86Y mutation on QN sensitivity has been explored by expression of the *pfmdr1 *gene in a heterologous system, *Xenopus *oocytes [33]. Substituting the asparagines (N) at position 86 for tyrosine (Y) resulted in a loss of QN transport ability. Since the site of QN action is in the food vacuole, [34,35] transport ability of the wild-type Pgh1 reduces drug concentration in the food vacuole, and consequently results in decreased QN susceptibility. This finding is compatible to that found in a few studies using parasites isolated from Southeast Asia, including the present study showing that the parasite isolates containing the *pfmdr1 *86Y allele showed more sensitive to QN [20,32]. However, a contrary result was shown in the study of parasite isolates from Kenya [17]. The decrease in QN susceptibility was associated with the *pfmdr1 *86Y allele in parasites harbouring the two DNNND repeats in the *pfnhe-1 *ms4760 microsatellite. Since reduced susceptibility to QN appear to be governed by a number of proteins whose contributions vary between strains. The contrasting findings might be explained by different variations in genetic background of parasites from different geographical areas. The influence of the *pfmdr1 *copy number on QN sensitivity has been confirmed by the study of Sidhu *et al*. using the knockdown strategy [12]. A few studies of parasite isolates from the Thai-Myanmar border also showed the influence of *pfmdr1 *copy number on *in vitro *QN sensitivity. However, this association has not been identified in some studies [21,22]. In this study, no association between the *pfmdr1 *copy number and *in vitro *QN sensitivity neither in parasites from the Thai-Myanmar nor the Thai-Cambodia border was identified.

In conclusion, the present study confirms the involvement of the *pfmdr1 *gene in QN sensitivity. Both N86Y and N1042D mutations significantly modulate *in vitro *response to QN. Although previous studies from Southeast Asia including Vietnam and China-Myanmar border have demonstrated that the *pfnhe-1 *gene is involved in QN sensitivity, the present study showed no association. This has raised doubt regarding the *pfnhe-1 *gene as to whether it could be used as the suitable marker for QN resistance in Thailand.

## Competing interests

The authors declare that they have no competing interests.

## Authors' contributions

TP and MM contributed to the conception and design of the study. NSu, NSi and RK performed *in vitro *susceptibility test and genotyping. TP, PT and MM analyzed the data and wrote the manuscript. All authors read and approved the final version that was submitted for publication.
